# Risk factors for acne vulgaris among rosacea patients: a cross-sectional study

**DOI:** 10.3389/fpubh.2026.1859962

**Published:** 2026-06-24

**Authors:** Shiyu He, Xintao Cen, Jiaojiao Zhong, Yaqian Zhu, Yang Guan, Jiarui Liu, Hongyun Guan, Xiaowen Hu, Min He, Ning Ning, Jiayu He, Zhiguang Zhao, Yanyan Li, Xiangzi Li

**Affiliations:** Shenzhen Center for Chronic Disease Control, Shenzhen Dermatology Hospital, Shenzhen Institute of Dermatology, Shenzhen, China

**Keywords:** acne vulgaris, comorbidity, correlation analysis, risk factors, rosacea

## Abstract

**Background:**

The prevalence of acne vulgaris among rosacea patients is significantly higher than that of the general population, but the risk factors for acne vulgaris among rosacea patients remain unclear.

**Objective:**

Explore the potential risk factors for the occurrence of acne vulgaris among rosacea patients, focusing on lifestyle, skincare habits, and dietary habits.

**Methods:**

A total of 300 rosacea patients were included. Lifestyle, skincare habits, and dietary habits were collected using a validated questionnaire survey. A Multivariate logistic regression model was employed to analyze risk factors associated with acne vulgaris. Further subgroup analysis and restricted cubic spline function was used to analyze the related risk factors.

**Results:**

The prevalence of acne vulgaris was 30.33% in 300 rosacea patients, which is significantly higher than the 14.83% observed in the general population. Multivariate logistic regression analysis revealed that sufficient sleep duration was a protective factor against acne vulgaris in rosacea patients [OR = 0.738 (0.589–0.926)] (*p* < 0.05), while the use of sunscreen products was identified as a risk factor [OR = 2.602 (1.083–6.249)], (*p* < 0.05). Additionally, restricted cubic spline analysis indicated a significant linear dose–response relationship between longer sleep duration and a lower risk of acne vulgaris (*P_overall_* < 0.05, *P_nonlinear_* = 0.86).

**Conclusion:**

Adequate sleep appears to be a protective factor, while frequent sunscreen use may increase the risk of acne vulgaris among rosacea patients. Tailored lifestyle recommendations may contribute to improved comorbidity management.

## 1 Introduction

Rosacea and acne vulgaris are two common chronic inflammatory dermatoses. Rosacea is characterized by vascular hyperreactivity and neurosensory hyperresponsiveness (1), whereas acne vulgaris primarily manifests as abnormal follicular keratinization and sebaceous gland dysfunction (2). Although these conditions exhibit significant differences in pathophysiology, susceptible populations, and clinical manifestations, conventional perspectives suggest their pathogenic mechanisms are mutually independent. However, the comorbidity of these two conditions is frequently observed in clinical practice. From the epidemiological investigation data of 2023 surveillance of chronic diseases and risk factors in Shenzhen (SCDRFS), we observed that the prevalence of acne among rosacea patients was significantly higher than that in the general population. This finding suggests that rosacea may constitute a potential risk factor for the development of acne.

Rosacea and acne vulgaris both involve cutaneous barrier dysfunction, aberrant innate immunity (Toll-like receptor 2 activation), pilosebaceous unit dysregulation, and microbial dysbiosis (3). Furthermore, lifestyle factors including dietary patterns (4), sleep–wake cycles (5, 6), and skincare habits (7) are recognized as significant contributors to the pathogenesis and progression of both diseases. In recent years, several studies have begun to investigate the impact of lifestyle factors on chronic inflammatory skin diseases. For instance, sleep deprivation may exacerbate skin inflammation by activating the hypothalamic–pituitary–adrenal axis and increasing cortisol levels (8). An epidemiological survey in the Chinese community shows that the prevalence of acne among women of childbearing age who have a sweet tooth and are sedentary is significantly higher than that in the control group (9). Similarly, the use of irritating skincare products can disrupt the skin barrier (10), triggering or worsening symptoms of acne and rosacea. Although these mechanisms have been partially validated, current research on how these factors specifically induce acne in rosacea patients remains limited, particularly regarding comprehensive assessments of specific risk factors. Existing studies have primarily focused on analyzing risk factors for individual diseases, without exploring in depth how lifestyle factors influence the risk of acne development in comorbid states.

Based on the above, present study intends to systematically evaluate the relationship between various lifestyle factors and the prevalence of acne, focusing on living habits, skincare habits, and dietary patterns, using a Multivariate logistic regression analysis approach and with rosacea patients as the study subjects. The findings of this study will fill the evidentiary gap in the field of comorbidity management for rosacea, providing theoretical support for the development of targeted lifestyle intervention strategies, such as sleep management guidelines, personalized skincare education, and dietary adjustment recommendations. Additionally, it lays the foundation for exploring multi-dimensional prevention and control models for skin comorbidities.

## 2 Methods

### 2.1 Participant recruitment

**Figure 1:**
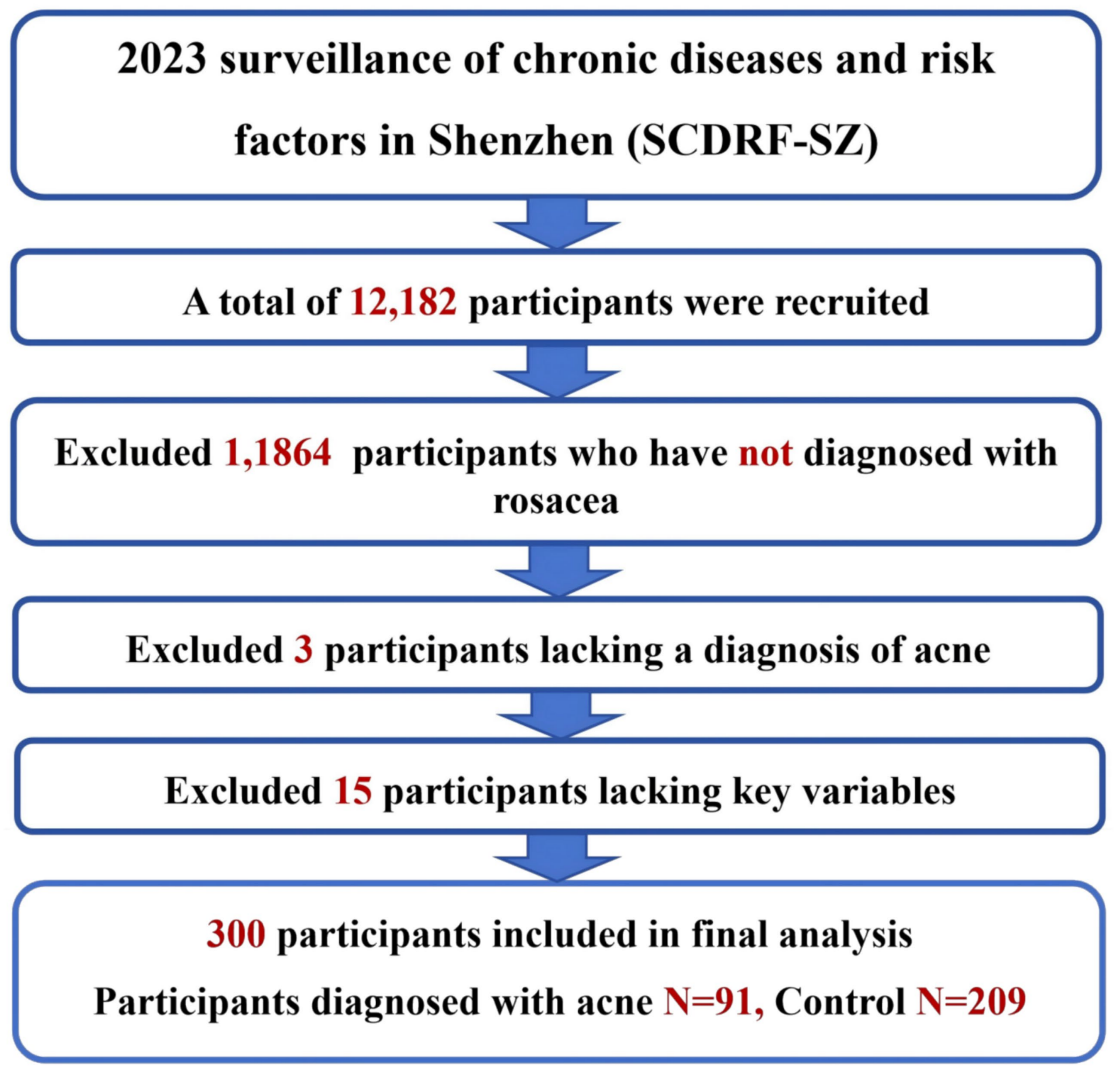
Schematic diagram of the participant enrollment process.

The SCDRFS project was employed a stratified sampling method to recruit 12,182 adult participants from the general population. After excluding individuals with missing data on key variables or study outcomes, participants diagnosed with rosacea by board-certified dermatologists were identified from the remaining dataset. A total of 300 individuals with rosacea were included in the present analysis (Figure 1). The research was approved by the Ethics Committee of Shenzhen Chronic Disease Prevention and Control Center (SZCCC-2023-034-01-PJ) and obtained written informed consent from all participants. The characteristics of these participants are presented in Table 1.

**Table 1:** Sociodemographic characteristics of rosacea participants.

Variables	Total (*N* = 300)	Acne (*N* = 91)	Non-acne (*N* = 209)	*p*-value
Age (years), mean±SD	43.82 ± 12.86	37.76 ± 10.61	46.46 ± 12.88	<0.0001
Gender, *n* (%)				0.0077
Male	120 (40.00)	26 (28.57)	94 (44.98)	
Female	180 (60.00)	65 (71.43)	115 (55.02)	
BMI (kg/m^2^), mean ± SD	24.25 ± 3.52 23.17 ± 3.19 24.72 ± 3.56	24.25 ± 3.52 23.17 ± 3.19 24.72 ± 3.56	24.25 ± 3.52 23.17 ± 3.19 24.72 ± 3.56	0.0004
Skintype, *n* (%)				0.0102
Dry	39 (13.09)	6 (6.59)	33 (15.94)	
Oil	129 (43.29)	52 (57.14)	77 (37.20)	
Normal	50 (16.78)	10 (10.99)	40 (19.32)	
Mix	62 (20.81)	19 (20.88)	43 (20.77)	
Sensitive	18 (6.04)	4 (4.40)	14 (6.76)	
Education, *n* (%)				0.0035
Tertiary education or above	173 (57.67)	41 (45.05)	132 (63.16)	
High school or below	127 (42.33)	50 (54.95)	77 (36.84)	
Nation, *n* (%)				0.0313
Han nationality	288 (96.00)	84 (92.31)	204 (97.61)	
Other nationality	12 (4.00)	7 (7.69)	5 (2.39)	
Marital status, *n* (%)				0.0049
Married or cohabitation	232 (77.33)	61 (67.03)	171 (81.82)	
Unmarried	68 (22.67)	30 (32.97)	38 (18.18)	
Insurance, *n* (%)				0.1011
Yes	286 (95.33)	84 (92.31)	202 (96.65)	
No	14 (4.67)	7 (7.69)	7 (3.35)	
Annual income (RMB)				0.6453
<50,000	28 (9.33)	10 (10.99)	18 (8.61)	
50,000 ~ 250,000	199 (66.33)	57 (62.64)	142 (67.94)	
≥250,000	73 (24.33)	24 (26.37)	49 (23.44)	
Smoking, *n* (%)				0.3018
Current	58 (19.33)	15 (16.48)	43 (20.57)	
Former	25 (8.33)	5 (5.49)	20 (9.57)	
Never	217 (72.33)	71 (78.02)	146 (69.86)	
Alcohol intake, *n* (%)				0.6551
Yes	187 (62.33)	55 (60.44)	132 (63.16)	
No	113 (37.67)	36 (39.56)	77 (36.84)	
Sleep time (hours/day), mean ± SD	7.96 ± 1.40	7.76 ± 1.33	8.05 ± 1.42	0.0921
Sedentary (hours/day), mean ± SD	6.84 ± 3.46	7.43 ± 3.19	6.58 ± 3.54	0.0504
Physical activity level, *n* (%)				0.0983
Low	49 (16.33)	21 (23.08)	28 (13.40)	
Moderate	140 (46.67)	41 (45.05)	99 (47.37)	
High	111 (37.00)	29 (31.87)	82 (39.23)	
Clean product, *n* (%)				0.0021
Yes	115 (38.33)	23 (25.27)	92 (44.02)	
No	185 (61.67)	68 (74.73)	117 (55.98)	
Moisturizing product, *n* (%)				0.0008
Yes	126 (42.00)	25 (27.47)	101 (48.33)	
No	174 (58.00)	66 (72.53)	108 (51.67)	
Mask, *n* (%)				0.0012
Yes	180 (60.00)	42 (46.15)	138 (66.03)	
No	120 (40.00)	49 (53.85)	71 (33.97)	
Sunscreen product, *n* (%)				0.0002
Yes	245 (81.67)	63 (69.23)	182 (87.08)	
No	55 (18.33)	28 (30.77)	27 (12.92)	
Cosmetic, *n* (%)				0.0073
Yes	225 (75.00)	59 (64.84)	166 (79.43)	
No	75 (25.00)	32 (35.16)	43 (20.57)	
Spicy food, *n* (%)				0.6974
Yes	183 (61.00)	54 (59.34)	129 (61.72)	
No	117 (39.00)	37 (40.66)	80 (38.28)	
Fruit, *n* (%)				0.6549
Yes	292 (97.33)	88 (96.70)	204 (97.61)	
No	8 (2.67)	3 (3.30)	5 (2.39)	
Milk, *n* (%)				0.1285
Yes	245 (81.67)	79 (86.81)	166 (79.43)	
No	55 (18.33)	12 (13.19)	43 (20.57)	
Beverages, *n* (%)				0.1286
Yes	185 (61.67)	62 (68.13)	123 (58.85)	
No	115 (38.33)	29 (31.87)	86 (41.15)	
Aquatic, *n* (%)				0.9029
Yes	281 (93.67)	85 (93.41)	196 (93.78)	
No	19 (6.33)	6 (6.59)	13 (6.22)	
Hypertension, *n* (%)				0.0348
Yes	62 (20.67)	12 (13.19)	50 (23.92)	
No	238 (79.33)	79 (86.81)	159 (76.08)	
Diabetes, *n* (%)				0.0258
Yes	31 (10.33)	4 (4.40)	27 (12.92)	
No	269 (89.67)	87 (95.60)	182 (87.08)	
Dyslipidemia, *n* (%)				0.1619
Yes	110 (36.67)	28 (30.77)	82 (39.23)	
No	190 (63.33)	63 (69.23)	127 (60.77)	

### 2.2 Clinical diagnosis and classification of rosacea and acne

The diagnosis of acne and rosacea was made face-to-face by a professional dermatologist on site. Rosacea was diagnosed according to the Chinese Guidelines for the Diagnosis and Treatment of Rosacea (2021 edition) (11). The required manifestations diagnostic criteria were as follows: (1) Persistent erythema on the cheeks, accompanied by episodic flushing and possible periodic exacerbation; or (2) Persistent erythema involving the perioral or nasal area, with possible periodic exacerbation. Supportive features: (1) flushing; (2) telangiectasia; (3) papules and pustules; (4) phymatous changes; and (5) ocular manifestations, including lid-margin telangiectasia, blepharitis, keratitis, conjunctivitis, or sclerokeratitis.

Rosacea was diagnosed when either of the following criteria was met: (1) Persistent erythema on the cheeks, regardless of the presence or absence of supportive features; or (2) Persistent erythema in the perioral or nasal area, together with at least one supportive feature, while excluding secondary causes of facial erythema.

Acne vulgaris was diagnosed if at least one of the following was present: (1) presence of current acne lesions; or (2) acne-related scarring, including moderate-to-severe scarring or hypertrophic/keloid scars, whereas acne-negative participants were required (1) no history of acne-related scarring and (2) no current acne lesions.

### 2.3 Covariate investigation

Demographic and lifestyle information was collected using a structured face-to-face questionnaire administered by trained public health professionals. The questionnaire covered socio-demographic characteristics, including age, sex, ethnicity, marital status, annual income level, educational attainment, dietary habits, skincare behaviors and lifestyle factors. Another section focused on health-related features, including blood glucose levels, BMI, hypertension and dyslipidemia. Height and weight were measured using standardized procedures by trained investigators. BMI was calculated as weight in kilograms divided by height in meters squared (kg/m^2^). Hypertension was defined based on the 2018 Guidelines of the European Society of Hypertension, which includes a self-reported diagnosis by a physician, the use of antihypertensive medications, or a systolic blood pressure ≥140 mmHg and/or diastolic blood pressure ≥90 mmHg. Participants’ blood pressure was measured after sitting and resting for 5 min, using automated equipment, three measurements were taken at one-minute intervals, and the average of the last two readings was used for analysis. Diabetes mellitus was defined according to the National Guidelines for the Prevention and Management of Diabetes in Primary Care (2022) as physician-diagnosed diabetes (12), current use of glucose-lowering medication, or fasting plasma glucose ≥7.0 mmol/L. Dietary habits were assessed using a retrospective self-administered questionnaire based on participants’ usual dietary intake over the past 1 year. Participants were asked to recall whether they consumed specific food groups in their daily life. The food groups included spicy foods, beverages, fruits, milk, and aquatic products. Skincare habits were assessed via self-report, including the frequency of use of cleansers, moisturizers, facial masks, sunscreen products, and cosmetics. Participants were asked: “How often do you use this product in your daily skincare routine?” Responses were categorized into four levels: almost never, occasionally, frequently, and daily. Smoking status was assessed using the question: “Do you currently smoke?” Smoking was defined as smoking at least one cigarette per day for a continuous or cumulative period of ≥6 months. Alcohol consumption was assessed using the question: “Do you drink alcohol?” Alcohol consumption was defined as the intake of any alcoholic beverages, including both commercially purchased and homemade drinks containing alcohol. Sedentary behavior was assessed using the question: “On a typical day, how much time do you usually spend sitting?” Sedentary behavior was defined as total sitting time at work or at home and during transportation, including activities such as desk work, car or bus travel, reading, playing cards, watching television, or using a computer, but excluding sleep time. Sleep duration was assessed using the question: “On average, how many hours do you sleep in a 24-hour period?”, referring to total daily sleep duration including naps. Physical activity level were assessed using the International Physical Activity Questionnaire, and based on total MET scores categorized into low, moderate, and high groups.

### 2.4 Statistical analysis

We utilized Analysis of Variance (ANOVA) for continuous variables and the Wald Chi-square test for categorical variables to compare participant characteristics, including gender, age, race, education level, marital status, income level, sleep duration, skin type, smoking status, drinking status, BMI, hypertension, diabetes and dyslipidemia. A multivariate logistic regression model was employed to estimate the odds ratios (ORs) and their 95% confidence intervals (CIs) between skincare habits, dietary habits, lifestyle habits and acne vulgaris among rosacea patients. After identifying relevant risk factors, restricted cubic spline functions were utilized to further analyze dose–response relationships or subgroup analysis.

All *p*-values were presented as two-sided, and statistical significance was determined based on a false discovery rate of less than 0.05. All statistical analyses were conducted using SAS software (version 9.4).

## 3 Result

### 3.1 Baseline characteristics of participants

A total of 300 participants were included in this study, with a mean age of 43.82 ± 12.86 years. Among them, 91 were diagnosed with acne vulgaris, corresponding to a prevalence rate of 30.33%, which was significantly higher than 14.83% in the general population (Supplementary Table S1). The mean BMI of the participants was 24.25 ± 3.52. Acne vulgaris was more common among younger, oil skin type, unmarried individuals, and those with lower education levels and incomes, static occupations and alcohol consumer (*p* < 0.05). Participants with acne had a lower prevalence of chronic conditions, including hypertension, diabetes, and dyslipidemia (*p* < 0.05) **(**Table 1).

### 3.2 Variables associated with acne in rosacea patients

After adjusting for all covariates, the results of the multivariate logistic regression analysis indicated that sleep duration with an OR = 0.738(0.589, 0.926) and the use of sunscreen products with an OR = 2.602(1.083, 6.249) were significantly associated with acne vulgaris among patients with rosacea (Table 2). Interestingly, independent risk factors for acne vulgaris in the general population, such as annual income level, nation, marital status, sedentary, and the use of cleansing products, did not show significant associations with acne vulgaris among rosacea patients (Supplementary Table S2).

**Table 2:** Multivariate logistic regression analysis of acne inducing factors in rosacea patients.

Variables	Odds ratio (95% CI)	*p*-value
Dietary habits		
Spicy food	0.664(0.352, 1.255)	0.207
Beverages	0.553(0.260, 1.176)	0.123
Fruit	0.515(0.074, 3.577)	0.501
Milk	1.439(0.596, 3.473)	0.417
Aquatic	0.815(0.238, 2.792)	0.745
Skin care habits		
Clean product	0.983(0.375, 2.580)	0.972
Moisturizing product	1.405(0.493, 4.005)	0.524
Mask	0.621(0.243, 1.584)	0.318
Sunscreen product	2.602(1.083, 6.249)*	**0.033**
Cosmetic	1.027(0.431, 2.450)	0.951
Living habits		
Smoking		
Never	Ref(1.00)	
Current smoker	1.624(0.578, 4.564)	0.358
Former smoker	0.948(0.265, 3.389)	0.934
Alcohol intake	0.853(0.421, 1.730)	0.659
Physical activity level		
Low	Ref(1.00)	
Moderate	1.308(0.514, 3.329)	0.573
High	1.040(0.505, 2.141)	0.915
Sleep time	0.738(0.589, 0.926)*	**0.008**
Sedentary	1.012(0.906, 1.130)	0.834

*Bold mean *p* < 0.05.

### 3.3 Dose–response and stratified analyses of key exposures

**Figure 2:**
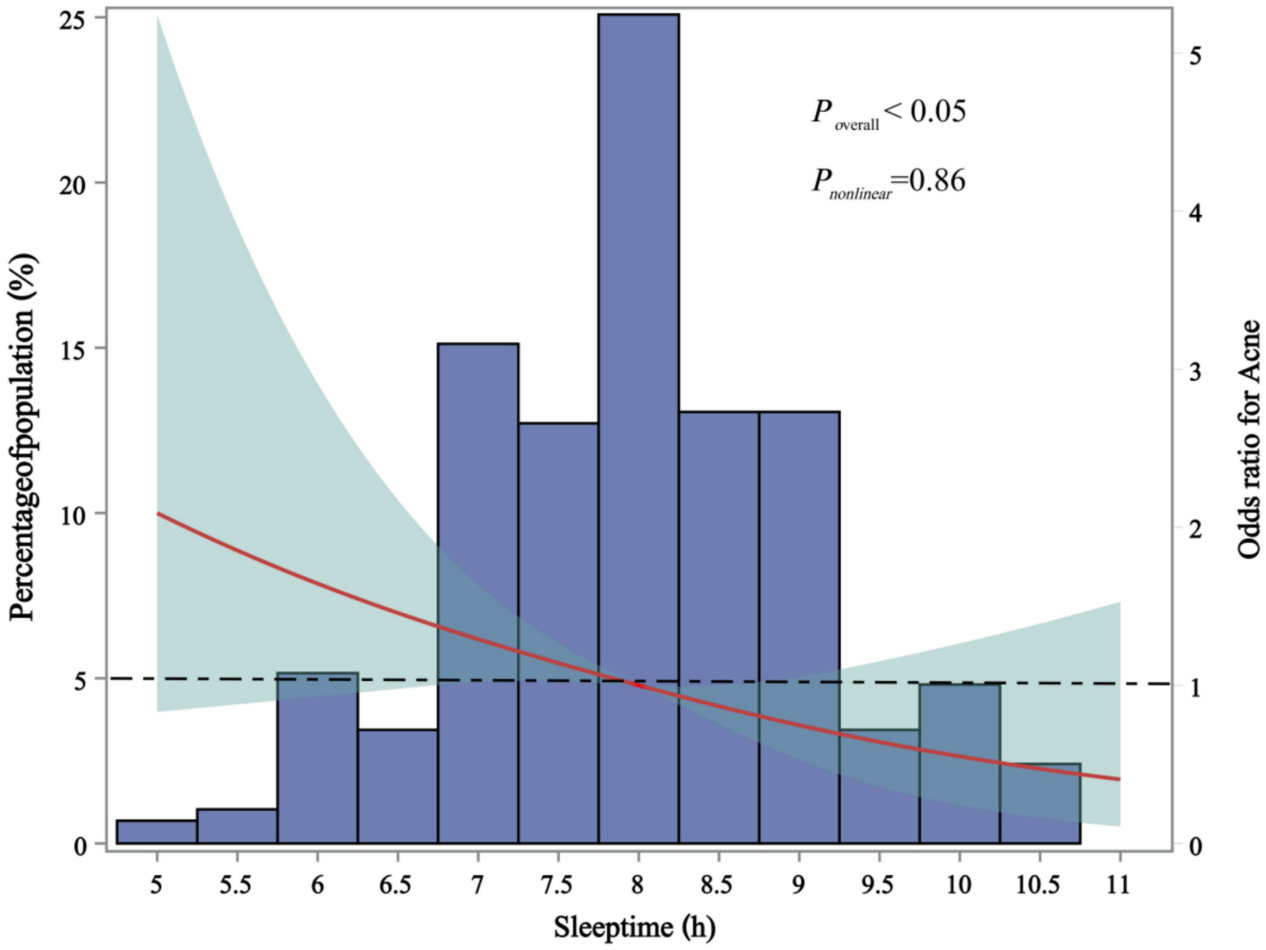
Dose–response curve between sleeptime and acne in restricted cubic spline model (*P*_nonlinear_ = 0.86). The variables were adjusted by age, gender, BMI, nation, marital status, education level, physical activity level, sedentary, hypertension, diabetes, cleanning product, moisturizing product, mask, sunscreen product, cosmetic. X-axis, self-reported sleep duration (hours/day). Left y-axis, distribution of study participants’ sleep duration corresponding to the blue histogram (%). Right y-axis, odds ratio (OR) for acne vulgaris, representing the association between sleep duration and acne occurrence. Blue histogram: distribution of sleep duration in the study population. Red solid line: estimated OR trend derived from the restricted cubic spline model. Green shaded area: 95% confidence interval (CI) of OR. Black dashed horizontal line: reference line at OR = 1.0.

We further stratified sunscreen product usage by frequency into never use, occasional use, frequent use, and daily use. A subgroup analysis was conducted to investigate the association between sunscreen usage frequency and the risk of acne vulgaris in rosacea patients. The results indicated that, compared to those who never used sunscreen products, the ORs for the risk of acne vulgaris in rosacea patients who used sunscreen occasionally, frequently, and daily were 1.408 (0.521, 3.806), 2.338 (0.522, 10.47), and 3.395 (1.153, 9.992), respectively (Table 3). Linear regression analysis of the dose–response relationship between sleep times and acne prevalence using a restricted cubic spline model showed a linear association (*P_overall_* < 0.05, *P_nonlinear_* = 0.86) (Figure 2).

**Table 3:** Multivariate logistic regression analysis of sunscreen product usage frequency and acne.

Variable	n	Model 1^a^	Model 2^b^	Model 3^c^	Model 4^d^
Sunscreen product usage frequency					
Never	202	1.00 (ref)	1.00 (ref)	1.00 (ref)	1.00 (ref)
Occasionally	43	1.942 (0.965, 3.907)	1.339 (0.593, 3.023)	1.202 (0.503, 2.869)	1.408 (0.521, 3.806)
Frequently	14	2.457 (0.812, 7.440)	1.600 (0.474, 5.397)	2.121 (0.554, 8.119)	2.338 (0.522, 10.47)
Daily	41	4.005 (1.982, 8.092)*	2.433 (1.056, 5.609)*	2.721 (1.082, 6.842)*	3.395 (1.153, 9.992)*

^a^Crude model.

^b^Adjusted for age, gender and BMI based on model 1.

^c^Further adjusted for nation, marital status, education level, health insurance, smoking status, alcohol intake, sleeptime, skintype based on model 2.

^d^Further adjusted for lifestyle, skincare habits and dietary habits based on model 3.

*mean *p* < 0.05.

## 4 Discussion

In this cross-sectional study involving 300 adult patients with rosacea, two modifiable lifestyle factors, sleep duration and the use of sunscreen products, were identified to be associated with acne vulgaris. Specifically, longer sleep duration was found to be a protective factor, whereas sunscreen use was correlated with an increased prevalence of acne vulgaris. Further subgroup analysis stratified by sunscreen application frequency revealed a dose-dependent relationship, with higher frequencies of sunscreen use associated with greater acne vulgaris prevalence, particularly among daily users who exhibited the highest risk.

There is still controversy over the relationship between sunscreen use and acne. Some studies suggest that sunscreen has a protective effect on acne, for examlpe, a clinical randomized controlled trial (*n* = 33) on acne patients who received multiple acne medications treatment indicated that daily use of sunscreen SPF30 with moisturizing properties could improve skin tolerance, assist in the treatment of patients, and reduce epidermal water loss (13). While a cross-sectional study on the skincare awareness of 450 patients with acne vulgaris found that individuals who used sunscreen had a significantly higher prevalence of mild acne, while the incidence of severe acne was significantly reduced (*p* < 0.001) (14). Others studies show that some chemical UV filters, such as benzophenone-4 and para-aminobenzoic acid derivatives, may induce cutaneous irritation, leading to varying degrees of erythematous papules and vesicles (15, 16). However, different from previous studies, this research focused on patients with rosacea, for whom the association between sunscreen use and acne risk may be attributed to the occlusive or comedogenic properties of certain sunscreen formulations. Although physical sunscreens containing zinc oxide and titanium dioxide are generally safer for sensitive skin, certain formulations may cause pore occlusion due to their thick texture (17). Furthermore, the incorporation of “water-resistant” or “sweat proof” ingredients in modern sunscreens often necessitates thorough cleansing. Incomplete removal of these residues may exacerbate follicular occlusion, while the required use of potent cleansers could compromise stratum corneum integrity. This detrimental effect is particularly pronounced in rosacea patients who exhibit pre-existing abnormalities in stratum corneum structure and skin barrier function (18). Interestingly, other variables considered risk factors for acne in the general population, such as ethnicity, marital status, annual household income, occupation, and the use of cleaning products, did not show significant associations with acne prevalence in the rosacea population. This suggests that the pathological process of rosacea may have changed the spectrum of common risk factors for acne to a certain extent. The inducing mechanism of acne in patients with rosacea is different from that in the general population and deserves further exploration.

The protective effect of longer sleep duration is consistent with previous research. Existing studies have shown that adequate sleep plays an important role in reducing systemic inflammation levels and promoting the repair of the skin barrier, which may have a positive impact on alleviating the severity of acne or rosacea (6). In addition, the disorder of the circadian rhythm is also regarded as a factor that aggravates the inflammatory response of rosacea. Studies have found that circadian rhythm disorders can affect the polarization of macrophages, causing them to polarize towards type M1, thereby promoting the expression of inflammatory factors such as tumor necrosis factor-*α*, interleukin-6, and interleukin-1β, which exacerbates the inflammatory response of skin lesions (19). Therefore, maintaining a regular schedule and getting sufficient sleep may play a key role in the treatment and management of rosacea.

This study emphasizes that when managing patients with rosacea complicated with acne, individualized skin care and lifestyle advice should be provided. Clinicians should be more cautious when recommending sun protection products. They should give priority to physical sun protection measures such as sun umbrellas and sun hats, or sun protection products that do not cause acne and are suitable for sensitive skin. At the same time, they should encourage patients to ensure adequate sleep to promote the improvement of acne.

## 5 Strengths and limitations

This study has the following strengths: First, the large sample size enabled us to identify a representative population of rosacea patients from the general population and comprehensively analyze risk factors associated with acne comorbidity. Second, the diagnosis of both rosacea and acne was performed by two board-certified dermatologists rather than relying on self-report or guideline-based criteria, significantly enhancing the reliability of subsequent analyses and conclusions. Third, this study innovatively investigated risk factors for acne in rosacea patients, along with subgroup analyses and linear association analyses, providing crucial evidence for developing scientific treatment and daily skincare guidelines for this patient population.

However, several limitations should be acknowledged: First, as a cross-sectional study, causality between risk factors and acne cannot be established, necessitating further validation through cohort studies. Second, several key exposures, including sleep duration and skincare behaviors were assessed using self-reported questionnaires, which may not capture the complexity of behavioral patterns (such as the composition of sunscreen, sleep quality, etc.), and introduce potential recall bias. Finally, the relatively low overall prevalence of rosacea in the general population may have limited the in-depth analysis of the relationship between risk factors and acne, potentially obscuring some associations. The observed associations require the use of more objective and comprehensive exposure assessment methods in the future and verification through prospective cohort studies to validate.

## 6 Conclusion

This study reveals that insufficient sleep time and frequent use of sunscreen products play an important role in the comorbidity of rosacea and acne vulgaris. It is worth noting that the risk factors for acne in patients with rosacea are different from those in the general population, suggesting that in clinical diagnosis, treatment and management of patients, there should be more targeted plans for the prevention and treatment of acne in patients with rosacea. In the future, it is necessary to further clarify the potential physiological mechanisms between the use of sunscreen products and sleep duration and the occurrence of acne through longitudinal research design and mechanism exploration and verify these associations in different populations.

## Data Availability

The original contributions presented in the study are included in the article/Supplementary material, further inquiries can be directed to the corresponding authors.

## References

[1] Geng RSQ Bourkas AN Mufti A Sibbald RG. Rosacea: pathogenesis and therapeutic correlates. J Cutan Med Surg. (2024) 28:178–89. doi: 10.1177/12034754241229365, 38450615 PMC11015710

[2] Cruz S Vecerek N Elbuluk N. Targeting inflammation in acne: current treatments and future prospects. Am J Clin Dermatol. (2023) 24:681–94. doi: 10.1007/s40257-023-00789-1, 37328614 PMC10460329

[3] Yang F Wang L Song D Zhang L Wang X Du D . Signaling pathways and targeted therapy for rosacea. Front Immunol. (2024) 15:1367994. doi: 10.3389/fimmu.2024.1367994, 39351216 PMC11439730

[4] Zujko-Kowalska K Masłowska J Knaś-Dawidziuk M Hamulka J Zujko ME. Dietary antioxidants may support cosmetic treatment in patients with rosacea. Antioxidants (Basel). (2024) 13:381. doi: 10.3390/antiox13030381, 38539914 PMC10968324

[5] Schrom KP Ahsanuddin S Baechtold M Tripathi R Ramser A Baron E. Acne severity and sleep quality in adults. Clocks Sleep. (2019) 1:510–6. doi: 10.3390/clockssleep1040039, 33089183 PMC7445853

[6] Wang Z Xie H Gong Y Ouyang Y Deng F Tang Y . Relationship between rosacea and sleep. J Dermatol. (2020) 47:592–600. doi: 10.1111/1346-8138.15339, 32291809

[7] Goh CL Wu Y Welsh B Abad-Casintahan MF Tseng CJ Sharad J . Challenges and real-world solutions for adoption of holistic skincare routine (cleansing, treatment, moisturization, and photoprotection) in acne, rosacea, atopic dermatitis, and sensitive skin: an expert consensus. J Cosmet Dermatol. (2024) 23:2516–23. doi: 10.1111/jocd.16396, 38853652

[8] Irwin MR. Sleep and inflammation: partners in sickness and in health. Nat Rev Immunol. (2019) 19:702–15. doi: 10.1038/s41577-019-0190-z, 31289370

[9] Wang YY Li SW Luo S Qin L Zeng X Li L . How to evaluate acne in reproductive-age women: an epidemiological study in Chinese communities. Biomed Res Int. (2019) 2019:1–5. doi: 10.1155/2019/6126808, 30854398 PMC6378077

[10] Khosrowpour Z Ahmad Nasrollahi S Samadi A Ayatollahi A Shamsipour M Rajabi-Esterabadi A . Skin biophysical assessments of four types of soaps by forearm in-use test. J Cosmet Dermatol. (2022) 21:3127–32. doi: 10.1111/jocd.14589, 34741581

[11] Gu H Hao F He W Jian D Jian Z Jiang X . Guidelines for the diagnosis and treatment of rosacea in China (2021 edition). Int J Dermatol Venereol. (2021) 4:199–209. doi: 10.1097/jd9.0000000000000197

[12] Chinese Diabetes Society; National Office for Primary Diabetes Care. National guidelines for the prevention and control of diabetes in primary care. Zhonghua Nei Ke Za Zhi. (2022) 61:249–62. doi: 10.3760/cma.j.cn112138-20220120-000063 35263966

[13] Baldwin H Santoro F Lachmann N Teissedre S. A novel moisturizer with high sun protection factor improves cutaneous barrier function and the visible appearance of rosacea-prone skin. J Cosmet Dermatol. (2019) 18:1686–92. doi: 10.1111/jocd.12889, 30803131 PMC6916358

[14] Hacinecipoğlu F Öner Ü. Assessing skincare awareness in acne vulgaris: a cross-sectional study. Adv Skin Wound Care. (2025) 39:E11–5. doi: 10.1097/ASW.0000000000000374, 41115133

[15] Kerr AC Niklasson B Dawe RS Escoffier A-m Krasteva M Sanderson B . A double-blind, randomized assessment of the irritant potential of sunscreen chemical dilutions used in photopatch testing. Contact Derm. (2009) 60:203–9. doi: 10.1111/j.1600-0536.2009.01516.x, 19338588

[16] Heurung AR Raju SI Warshaw EM. Adverse reactions to sunscreen agents: epidemiology, responsible irritants and allergens, clinical characteristics, and management. Dermatitis. (2014) 25:289–326. doi: 10.1097/DER.0000000000000079, 25384223

[17] Liang Y Simaiti A Xu M Lv S Jiang H He X . Antagonistic skin toxicity of co-exposure to physical sunscreen ingredients zinc oxide and titanium dioxide nanoparticles. Nanomaterials (Basel, Switzerland). (2022) 12:769. doi: 10.3390/nano12162769, 36014634 PMC9414962

[18] Logger JGM Driessen RJB de Jong E van Erp PEJ. Value of GPSkin for the measurement of skin barrier impairment and for monitoring of rosacea treatment in daily practice. Skin Res Technol. (2021) 27:15–23. doi: 10.1111/srt.12900 32573826 PMC7984125

[19] Tu Y Yang Z He Y Wang T Hua P Yao Q . Circadian rhythm disruption promotes M1 macrophages polarization exacerbating the inflammatory response in rosacea. Arch Dermatol Res. (2025) 317:658. doi: 10.1007/s00403-025-04060-x, 40167798

