# Supplementary material for: Risk factors for acne vulgaris among rosacea patients: a cross-sectional study
Source: Front Public Health. 2026 Jun 24;14:1859962. doi: 10.3389/fpubh.2026.1859962 (PMC13341721; doi:10.3389/fpubh.2026.1859962)
Supplement: Supplementary file 1 [file Table_1.DOCX]

**Table S1.** Characteristics of Shenzhen adults according to acne.

| **Variables** | **Total (N=11825)** | **Acne (N=1754)** | **Non-acne (N=10071)** | ***P*-Value** |
| --- | --- | --- | --- | --- |
| **Age (years), mean±SD** | 45.43±12.75 | 33.71±8.06 | 47.47±12.31 | <.0001 |
| **Gender, n (%)** |  |  |  | 0.0003 |
| Male | 5520 (46.68) | 749 (42.70) | 4771 (47.37) |  |
| Female | 6305 (53.32) | 1005 (57.30) | 5300 (52.63) |  |
| **BMI(kg/m²), mean ± SD** | 24.22±3.58 | 23.44±3.85 | 24.36±3.51 | <.0001 |
| **Skintype, n (%)** |  |  |  | <.0001 |
| Dry | 2158 (18.25) | 186 (10.60) | 1972 (19.58) |  |
| Oil | 3825 (32.35) | 953 (54.33) | 2872 (28.52) |  |
| Normal | 4557 (38.54) | 318 (18.13) | 4239 (42.09) |  |
| Mix | 1156 (9.78) | 284 (16.19) | 872 (8.66) |  |
| Sensitive | 49 (0.41) | 8 (0.46) | 41 (0.41) |  |
| **Education, n (%)** |  |  |  | <.0001 |
| Tertiary education or above | 7367 (62.30) | 717 (40.88) | 6650 (66.03) |  |
| High school or below | 4458 (37.70) | 1037 (59.12) | 3421 (33.97) |  |
| **Nation, n (%)** |  |  |  | <.0001 |
| Han nationality | 11318 (95.71) | 1630 (92.93) | 9688 (96.20) |  |
| Other nationality | 507 (4.29) | 124 (7.07) | 383 (3.80) |  |
| **Marital status, n (%)** |  |  |  | <.0001 |
| Married | 9531 (80.60) | 1076 (61.35) | 8455 (83.95) |  |
| Unmarried | 2294 (19.40) | 678 (38.65) | 1616 (16.05) |  |
| **Insurance, n (%)** |  |  |  | 0.0174 |
| Yes | 11141 (94.22) | 1674 (95.44) | 9467 (94.00) |  |
| No | 684 (5.78) | 80 (4.56) | 604 (6.00) |  |
| **Annual income, (RMB)** |  |  |  | <.0001 |
| <50,000 | 1408 (11.91) | 152 (8.67) | 1256 (12.47) |  |
| 50000~250,000 | 7780 (65.79) | 1167 (66.53) | 6613 (65.66) |  |
| ≥250,000 | 2637 (22.30) | 435 (24.80) | 2202 (21.86) |  |
| **Smoking, n (%)** |  |  |  | 0.0001 |
| Current | 2521 (21.32) | 366 (20.87) | 2155 (21.40) |  |
| Former | 682 (5.77) | 64 (3.65) | 618 (6.14) |  |
| Never | 8622 (72.91) | 1324 (75.48) | 7298 (72.47) |  |
| **Alcohol intake, n (%)** |  |  |  | <.0001 |
| Yes | 7080 (59.87) | 1140 (64.99) | 5940 (58.98) |  |
| No | 4745 (40.13) | 614 (35.01) | 4131 (41.02) |  |
| **Sleep time**  **(hours/day), mean ± SD** | 8.05±1.28 | 8.19±1.18 | 8.03±1.29 | <.0001 |
| **Sedentary**  **(hours/day), mean ± SD** | 6.39±3.35 | 7.82±3.33 | 6.14±3.30 | <.0001 |
| **Physical activity Level, n (%)** |  |  |  | <.0001 |
| Low | 1987 (16.80) | 395 (22.52) | 1592 (15.81) |  |
| Moderate | 4969 (42.02) | 853 (48.63) | 4116 (40.87) |  |
| High | 4869 (41.18) | 506 (28.85) | 4363 (43.32) |  |
| **Clean product, n (%)** |  |  |  | <.0001 |
| Yes | 5325 (45.03) | 366 (20.87) | 4959 (49.24) |  |
| No | 6500 (54.97) | 1388 (79.13) | 5112 (50.76) |  |
| **Moisturizing product, n (%)** |  |  |  | <.0001 |
| Yes | 6156 (52.06) | 637 (36.32) | 5519 (54.80) |  |
| No | 5669 (47.94) | 1117 (63.68) | 4552 (45.20) |  |
| **Mask, n (%)** |  |  |  | <.0001 |
| Yes | 8045 (68.03) | 886 (50.51) | 7159 (71.09) |  |
| No | 3780 (31.97) | 868 (49.49) | 2912 (28.91) |  |
| **Sunscreen product, n (%)** |  |  |  | <.0001 |
| Yes | 10019 (84.73) | 1342 (76.51) | 8677 (86.16) |  |
| No | 1806 (15.27) | 412 (23.49) | 1394 (13.84) |  |
| **Cosmetic, n (%)** |  |  |  | <.0001 |
| Yes | 9805 (82.92) | 1219 (69.50) | 8586 (85.25) |  |
| No | 2020 (17.08) | 535 (30.50) | 1485 (14.75) |  |
| **Spicy food, n (%)** |  |  |  | <.0001 |
| Yes | 7265 (61.44) | 1243 (70.87) | 6022 (59.80) |  |
| No | 4560 (38.56) | 511 (29.13) | 4049 (40.20) |  |
| **Fruit, n (%)** |  |  |  | 0.9863 |
| Yes | 11556 (97.73) | 1714 (97.72) | 9842 (97.73) |  |
| No | 269 (2.27) | 40 (2.28) | 229 (2.27) |  |
| **Milk, n (%)** |  |  |  | <.0001 |
| Yes | 9119 (77.12) | 1463 (83.41) | 7656 (76.02) |  |
| No | 2706 (22.88) | 291 (16.59) | 2415 (23.98) |  |
| **Beverages, n (%)** |  |  |  | <.0001 |
| Yes | 7116 (60.18) | 1399 (79.76) | 5717 (56.77) |  |
| No | 4709 (39.82) | 355 (20.24) | 4354 (43.23) |  |
| **Aquatic, n (%)** |  |  |  | 0.3819 |
| Yes | 11110 (93.95) | 1656 (94.41) | 9454 (93.87) |  |
| No | 715 (6.05) | 98 (5.59) | 617 (6.13) |  |
| **Hypertension, n (%)** |  |  |  | <.0001 |
| Yes | 2782 (23.53) | 182 (10.38) | 2600 (25.82) |  |
| No | 9043 (76.47) | 1572 (89.62) | 7471 (74.18) |  |
| **Diabetes, n (%)** |  |  |  | <.0001 |
| Yes | 970 (8.20) | 64 (3.65) | 906 (9.00) |  |
| No | 10855 (91.80) | 1690 (96.35) | 9165 (91.00) |  |
| **Dyslipidemia, n (%)** |  |  |  | <.0001 |
| Yes | 4582 (38.75) | 495 (28.22) | 4087 (40.58) |  |
| No | 7243 (61.25) | 1259 (71.78) | 5984 (59.42) |  |

**Table S2.** Multivariate logistic regression analysis of acne inducing factors in general population.

| **Variables** | **Odds ratio (95% CI)** | ***P*-value** |
| --- | --- | --- |
| Marital status | **0.723(0.629, 0.830)** | **<.0001** |
| Education level | **0.788(0.693, 0.896)** | **<.0001** |
| Nation | **0.685(0.541, 0.867)** | **0.002** |
| **Dietary habit** |  |  |
| Spicy food | 1.019(0.900, 1.155) | 0.765 |
| Beverages | **1.295(1.124, 1.492)** | **<0.001** |
| Fruit | 0.761(0.512, 1.131) | 0.177 |
| Milk | 0.975(0.837, 1.137) | 0.750 |
| Aquatic | 0.934(0.724, 1.205) | 0.599 |
| **Skin care habits** |  |  |
| Clean product | **1.639(1.395, 1.925)** | **<.0001** |
| Moisturizing product | 1.012(0.845, 1.213) | 0.896 |
| Mask | 1.169(0.992, 1.378) | 0.063 |
| Sunscreen product | 0.953(0.809, 1.122) | 0.565 |
| Cosmetic | 1.134(0.961, 1.337) | 0.136 |
| **Living habits** |  |  |
| Smoking |  |  |
| Never | 1.057(0.891, 1.252) | 0.526 |
| Current smoker | 1.040(0.765, 1.412) | 0.804 |
| Former smoker | 0.991(0.872, 1.127) | 0.891 |
| Alcohol intake |  |  |
| Physical activity level | 1.108(0.939, 1.306) | 0.224 |
| low | 1.077(0.941, 1.232) | 0.281 |
| Moderate | **1.051(1.002, 1.102)** | **0.040** |
| High | **1.026(1.007, 1.045)** | **0.008** |
